# Mix and wait – a relaxed way for synthesizing ZIF-8†

**DOI:** 10.1039/d2ra00740a

**Published:** 2022-03-22

**Authors:** Nikita Gugin, Jose A. Villajos, Ines Feldmann, Franziska Emmerling

**Affiliations:** Federal Institute for Materials Research and Testing (BAM) Richard-Willstätter-Str. 11 12489 Berlin Germany franziska.emmerling@bam.de; Department of Chemistry, Humboldt Universität zu Berlin Brook-Taylor-Str. 12489 Berlin Germany

## Abstract

Herein we report the synthesis of a zeolitic imidazolate framework (ZIF-8) by an easy “mix and wait” procedure. In a closed vial, without any interference, the mixture of 2-methylimidazole and basic zinc carbonate assembles into the crystalline product with approx. 90% conversion after 70 h. The reaction exhibits sigmoidal kinetics due to the self-generated water which accelerates the reaction.

## Abstract 2

Herein we report the synthesis of a zeolitic imidazolate framework (ZIF-8) by an easy “mix and wait” procedure.

## Graphical abstract

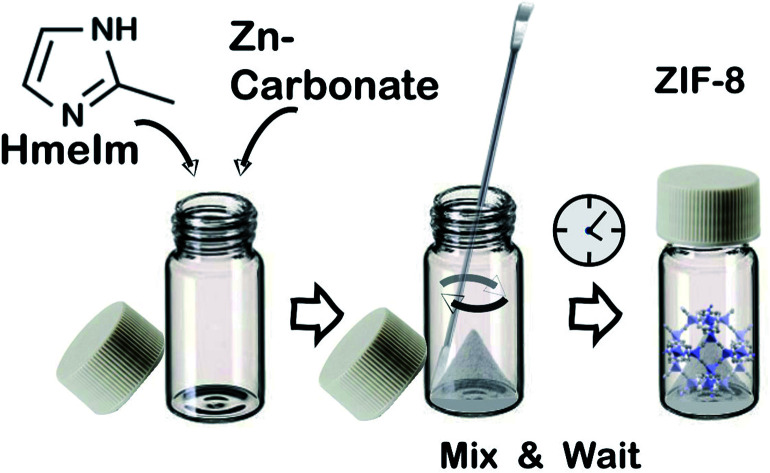

The synthesis and development of metal–organic frameworks (MOFs) have grown very rapidly in the last decades, driven by numerous potential applications in different areas due to their outstanding porosity and chemical versatility.^1–6^ Zeolitic imidazolate frameworks (ZIFs) represent a subfamily of MOFs, consisting of tetrahedrally coordinated metal nodes connected by imidazolate ligands.^7–9^ A prominent member of the ZIF subfamily is ZIF-8 or Zn(meIm)_2_ (meIm = deprotonated 2-methylimidazole), which possesses high thermal, chemical, and mechanical stabilities.^7^ ZIF-8 and doped variants with different metals have been studied for many applications, *e.g.* gas separation,^10^ photocatalysis,^11^ drug delivery,^12^ and wastewater treatment.^13^

Although a great variety of ZIF-8 synthetic approaches has been well-documented, these procedures always involve the use of solvents,^14,15^ steam,^16^ supercritical CO_2_,^17^ mechanical impact,^18–24^ pressure,^25^ or elevated temperatures.^20,26^ Here we report a simple “mix and wait” synthetic procedure using as reagents basic zinc carbonate (ZnCarb, [ZnCO_3_]_2_·[Zn(OH)_2_]_3_) and 2-methylimidazole (HmeIm, C_4_H_6_N_2_). Remarkably, ZIF-8 can be readily synthesized just by bringing the solid precursors in contact in a closed vial.

ZIF-8 was obtained *via* direct reaction between HmeIm and ZnCarb in a HmeIm/Zn molar ratio of 2 : 1. For this, the reagents were thoroughly mixed, sealed in 8 mL glass vials, and kept at ambient temperature for different periods (see the ESI† for details).

Without any further interference, the mixture in the closed vial reacts, forming highly crystalline ZIF-8 after 16 h (Fig. 1 and S1 in the ESI†). The powder X-ray diffraction (PXRD) patterns show that the reactants conversion increases for longer reaction times, as evidenced by the decrease in the relative intensity of HmeIm reflections accompanied by a decrease in width of the reflection at 2*θ* = 13°, which is a convolution of reflections of ZIF-8 and ZnCarb. After 7 days, no reflections of the reactants can be observed in the PXRD pattern. Instead, a reflection at 2*θ* ≈ 12° appears after 7 days, corresponding to a previously reported carbonate-based side phase with formula Zn(meIm)_2_·ZnCO_3_.^19^ This side-product results from the reaction between initially formed ZIF-8 and CO_2_, and its content increases with the contact time.

**Fig. 1:**
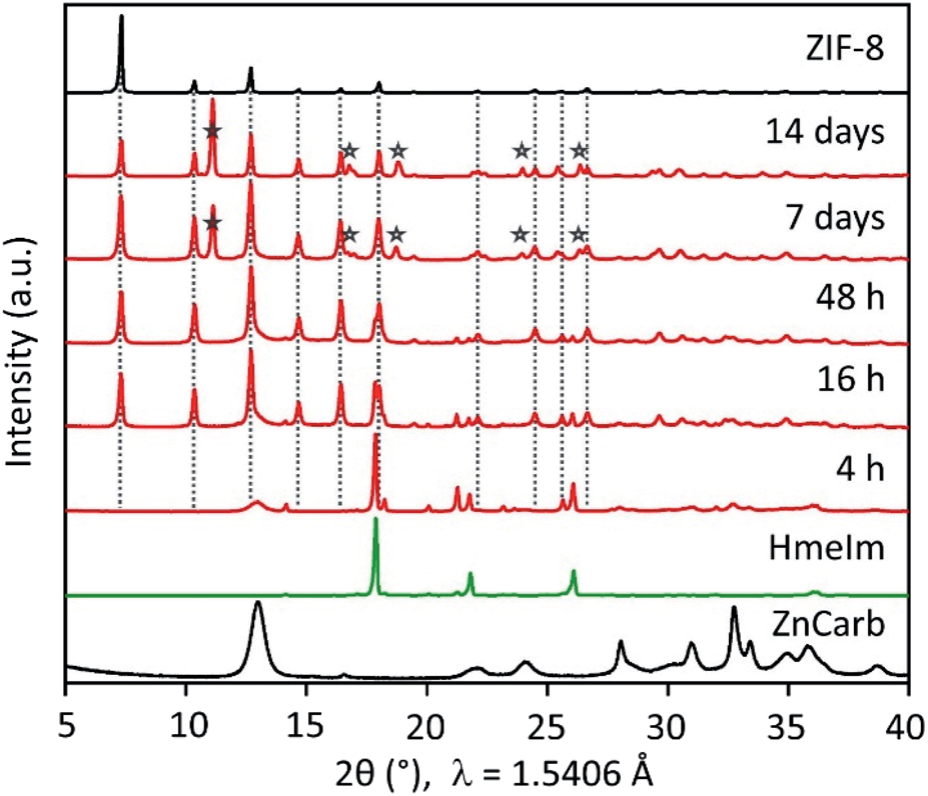
PXRD patterns of HmeIm (green line), ZnCarb (black line on bottom), reference ZIF-8 (black line on top), and products prepared at different times (red lines). Characteristic reflections of the side phase Zn(meIm)_2_·ZnCO_3_ are marked by asterisks (★).

The reaction rate increases by simply shaking the reactant mixture on a laboratory mini shaker with the circular motion of the agitation table. PXRD pattern of samples treated this way shows no reflections corresponding to HmeIm after 46 h (see Fig. S2 in the ESI†). Notably, the comparison between different experiments performed under the same conditions (see Fig. S1 and S2 in ESI†) demonstrates a surprisingly good reproducibility. In contrast to the reaction in closed vials, the mixture of the reactants in the open vial did not produce an appreciable transformation to ZIF-8 even after one month (Fig. S1 in ESI†). When the metal source different from ZnCarb is used (*i.e.* zinc acetate dihydrate, zinc nitrate hexahydrate, or zinc hydroxide), the ZIF-8 formation also does not occur (Fig. S3 in ESI†).

To follow the reaction mechanism and kinetics for the ZIF-8 formation, we performed *in situ* Raman spectroscopy experiments (see Section 3.2 in ESI†) monitoring the “mix and wait” reaction between HmeIm and ZnCarb. The experiment was conducted for 48 h collecting Raman spectra every 15 min. The jar contribution subtraction, baseline correction, and normalisation were performed using a script for basic analysis and visualisation of *in situ* Raman monitoring data.^27^ The time-resolved 2D Raman spectra (Fig. 2a) show only bands attributed to HmeIm at the beginning of the reaction. The first Raman bands of ZIF-8 appear after an induction period of 1 h and 45 min. Depletion of HmeIm can be followed by changes in the intensity of Raman bands at 1481, 1127, and 681 cm^−1^, while the formation of ZIF-8 is evident from the increase in the intensity of characteristic bands at *ν*_*ν*C–N+N–Hwag_ = 1498 cm^−1^, *ν*_C–Hwag_ = 1461 cm^−1^, and *ν*_*ν*C–N_ = 1147 cm^−1^, without formation of any reaction intermediates (assignment based on the ref. 28; *ν* – stretching, wag – wagging). HmeIm and ZIF-8 bands coexist in the final product (Fig. 2a, red), indicating that the reaction is not complete. After calibration of the Raman signals (see Section 3.3 in ESI†), the conversion was estimated as 65% after 48 h. A kinetic profile (Fig. 2b) is obtained by plotting intensities at the maximum of the peaks 1481 cm^−1^ (HmeIm) and 1461 cm^−1^ (ZIF-8) *versus* time. The kinetics are sigmoidal, with an induction period followed by a steep increase in reaction rate finalizing with the reaction equilibrium, as it is typically reported for autocatalytic reactions.^29^ An example is the synthesis of ZIF-8 from the ZnO/HmeIm mixture following a liquid-assisted grinding procedure (LAG).^30^

**Fig. 2:**
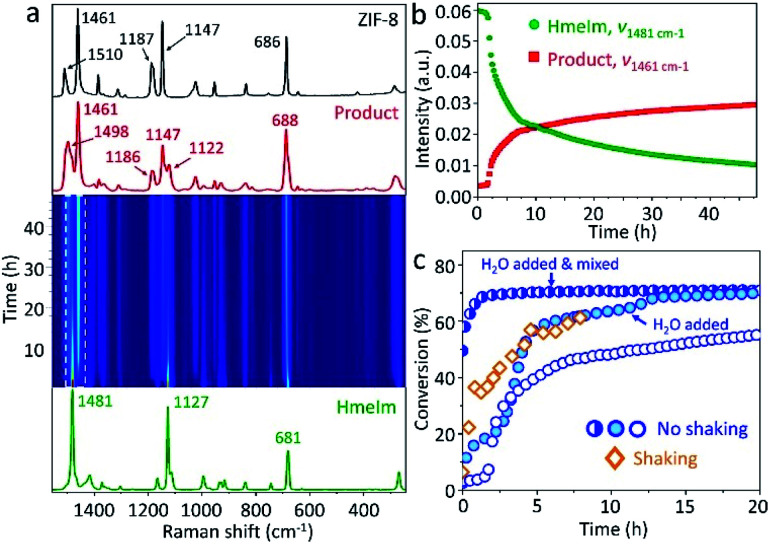
(a) Time-resolved 2D Raman spectra of ZIF-8 self-assembly in the range between 1380 and 1540 cm^−1^. The Raman spectrum of HmeIm (green line) is given below the 2D plot, and Raman spectra of the product (red line) and reference ZIF-8 (black line) are given above. Reaction profile obtained (b) by plotting intensities at the peak maximum *versus* time for HmeIm and product and (c) by plotting estimated conversion *versus* time for no shaking and shaking experiments with and without H_2_O addition.

We considered two factors that could potentially give rise to sigmoidal kinetics: (i) the static nature of the reaction, limited by the solid-state diffusion of the reagents during the induction phase, (ii) H_2_O formed as a byproduct could act as a self-generated solvent for HmeIm, thus, promoting the contact between reactants when enough water has accumulated in the system. The shaking experiment (Fig. 2c, diamonds) has no induction period and reaches a conversion of 60% after 8 h, enhancing the reaction rate. This contrasts with the 1 h and 45 min of induction time for the non-shaken reaction (Fig. 2c, empty circles) which reaches 47% of conversion after 8 h. To test whether H_2_O could be responsible for the kinetics, we performed two experiments with water added at the start of the experiment. In the first case, 50 μL of H_2_O was added to the reagents and mixed with a spatula for 1 min. The corresponding profile shows no induction period (Fig. 2c, half-filled circles), and the reaction starts from a 50% conversion in the first recorded spectrum (here, we should note that the time between mixing and start of the experiment is *ca.* 4 min). Just 1.5 h later, the conversion is 69%, and the reaction reaches equilibrium after 8 h with a conversion of 71%. When the same amount of H_2_O was added to the bottom of the PMMA jar, and the dry reaction mixture was placed above, the recorded profile exhibits two acceleration regions of the reaction rate without induction period (Fig. 2c, filled circles), reaching conversions of 60 and 68% after 6 and 13 h, respectively. Similar to the first water-assisted experiment, the reaction achieves equilibrium *ca.* 70% of conversion after 20 h. The mixture prepared under dry argon atmosphere and kept in the closed vial did not produce an appreciable transformation to ZIF-8 after 48 h (Fig. S3 in ESI†).

In general, we have demonstrated that shaking and water addition increase the reaction rate and minimize the induction period, indicating that the slow reactant diffusion along with the self-generated water as a byproduct may be directly responsible for the observed kinetics. Another reason for the sigmoidal kinetics may result from a physical form of the reaction mixture: as the reaction progresses, the initial mixture of reagents transforms from free-flowing powder to a cohesive state which returns to a free-flowing powder in a short time after opening the vial. This change in mixture rheology likely leads to a change in chemical kinetics (*e.g.*, due to a change in molecular diffusion rate). Thus, the cohesive state formation can also explain the sigmoidal kinetic profile, as it was observed for a reported Knoevenagel condensation.^31^

To perform further characterisation of the reaction product, we chose the sample obtained by keeping ZnCarb and HmeIm in contact for 70 h. The as-synthesized sample has no PXRD signals related to reactants and only a tiny peak of the side phase (Fig. 3a). In contrast to PXRD measurements, thermogravimetric analysis (TGA) shows a weight loss between 120 and 200 °C (Fig. S4 in ESI†), which we ascribe to evaporation of remaining HmeIm. Removal of unreacted ligand was performed according to the reported procedure by washing and sonicating the as-synthesized powder in 50 mL of ethanol three times with subsequent drying at 60 °C overnight.^21^ TGA confirms that HmeIm was removed from the product (Fig. S4 in ESI†). The material remains its crystal structure after washing (Fig. 3a), yet exhibiting a higher relative intensity of the reflection at 2*θ* ≈ 7° compared to the non-washed sample. This could indicate that the as-synthesized sample has some linker molecules in the pores, which reduce the diffraction intensity of the largest interplanar distance within the crystal. Meanwhile, ZnCarb cannot be removed using the applied washing procedure. Thus, the TGA weight loss related to the ZnCarb thermal decomposition at ∼270 °C (Fig. S4 in ESI†) can be used to estimate the ZnCarb-to-ZIF-8 conversion rate since ZIF-8 does not induce weight loss at this temperature. The theoretical weight loss for the decomposition of ZnCarb (549.0 g mol^−1^) into ZnO (81.4 g mol^−1^) is 25.9%. Hence the ZnCarb-to-ZIF-8 conversion rate can be calculated using the formula conversion rate = 100 × (1 − weight loss (%)/25.9 (%)) as 92%. The final weight loss step recorded at 380–530 °C was ascribed to the decomposition of ZIF-8 into ZnO.

**Fig. 3:**
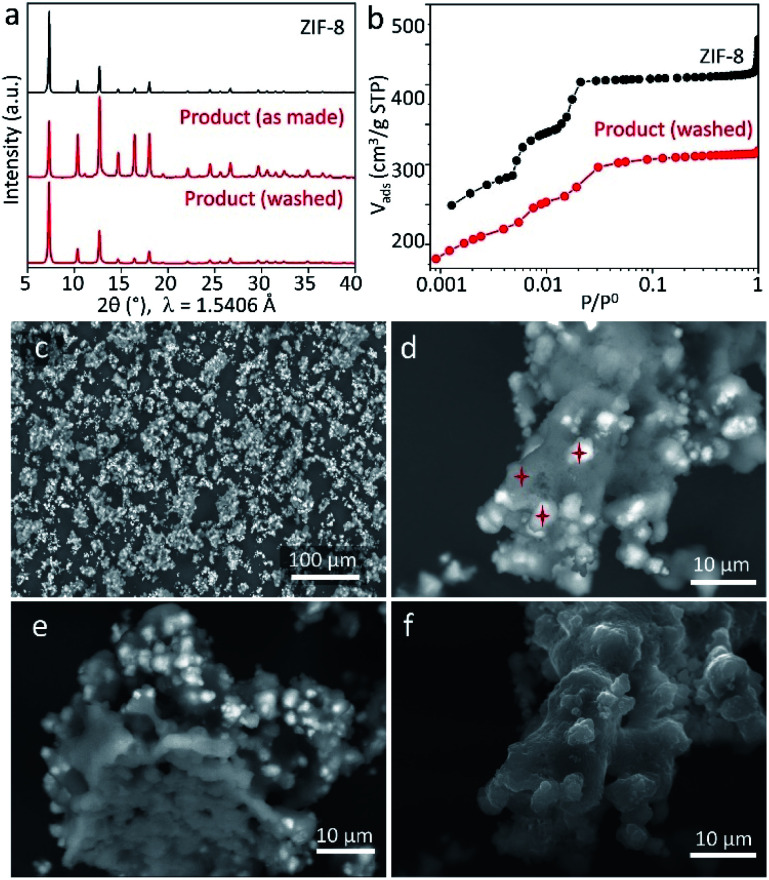
Characterisation of the product synthesized after 70 h. (a) PXRD patterns of as-made and ethanol-washed products (red) in comparison with the reference ZIF-8 pattern (black). (b) Nitrogen adsorption isotherm at 77 K of the washed product (red) compared to the isotherm of the reference ZIF-8 (black). (c–f) SEM pictures of the as-synthesized product using BSE (c–e) and SE (f) detectors. Red stars indicate the spots where EDX spectra were recorded (d).

The type I N_2_ adsorption isotherm of the washed sample indicates a microporous structure according to the IUPAC classification.^32^ The shape of the isotherm at small relative pressure *p*/*p*_0_ plotted in logarithmic scale is very similar to that of the reference ZIF-8 (Fig. 3b), indicating a similar microporous structure. The small differences in the N_2_ uptake for each adsorption step could be attributed to ZnCarb leftovers occluded in pores. The calculated Brunauer–Emmett–Teller (BET) surface area and total pore volume (at *p*/*p*_0_ ≈ 0.9) are 1304 m^2^ g^−1^ and 0.48 cm^3^ g^−1^, lower than the 1734 m^2^ g^−1^ and 0.64 cm^3^ g^−1^ of the reference ZIF-8, which can be due to the mass addition of unreacted ZnCarb and a possible partial pore-blocking effect.

Scanning electron microscopy (SEM) micrographs obtained using secondary (SE) and backscattered electrons (BSE) confirm the presence of ZnCarb leftovers as dispersed irregular agglomerates with non-homogeneous brightness (Fig. 3c–f and S5 in ESI†). The elemental composition in different spots throughout bright and dark regions (marked stars in Fig. 3d) was measured by energy-dispersive X-ray spectroscopy (EDX) for Zn, N, and O (Table S1 in ESI†). Due to the raw-sample's surface morphology, the elemental composition obtained must be considered as a rough estimate. Quantitative comparisons of the results acquired under the same experimental conditions are more realistic than the absolute values. In the dark spots, the average atomic Zn/N ratio is 0.2, close to the value of 0.25 for pure ZIF-8. However, EDX reveals a small but significant contribution of oxygen, indicating that minor amounts of ZnCarb are also present in the dark regions. The average Zn/O ratio in bright regions (∼0.5) is similar to that for ZnCarb (0.4) and considerably lower than that in dark regions (∼2.0). The significant content of nitrogen in the bright spots, which should not appear in the ZnCarb region, along with the relatively similar surface morphology of bright and dark regions (Fig. 3f, taken with the SE detector), suggests that ZnCarb particles are not externally attached to the surface of aggregates but rather hidden under a thin outer layer of ZIF-8. The internal composition of the aggregates is seen on the micrograph of the apparently “broken” particle and shows a relatively homogeneous composition without bright spots (Fig. 3e, taken with BSE detector). Corresponding SEM images recorded after shaking the mixtures for 4, 16, and 46 h show that the agglomeration of particles increases with time, still maintaining similar proportion of bright areas (Fig. S6†). The irregular morphology observed herein is different from cubic and rhombic crystals with a size of 50–2000 nm typically obtained in methanol solution.^33,34^

Based on our results, we suggest that the reaction of ZIF-8 self-assembly occurs as shown schematically in Fig. 4. The reaction is probably initiated on the contact surface of ZnCarb and HmeIm with the aid of ambient moisture. When the reaction is initiated, it produces water as the by-product, which accumulates in the system and accelerates ZIF-8 formation. Generated H_2_O partially dissolves HmeIm forming a “solution-like” phase around ZnCarb particles. The ZIF-8 crystallisation begins at the solid–solution interface and proceeds deeper in the ZnCarb agglomerates as the reaction progresses and more HmeIm/water solution diffuses to the ZnCarb cores. Since the conversion induces a volume expansion of the solid, adjacent particles agglomerate over time. During their growing up, the agglomerates could collect surrounding unreacted ZnCarb on their surface, thus fueling the reaction and further growth.

**Fig. 4:**
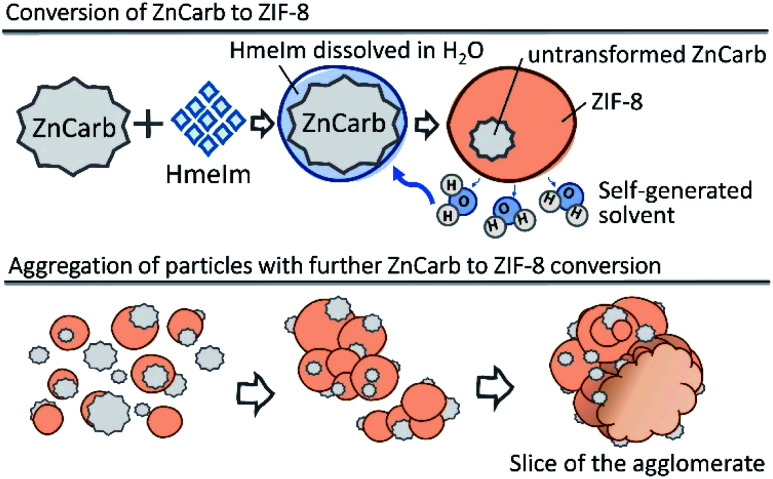
Schematic diagram of ZIF-8 self-formation. Upper panel: formation of ZIF-8, lower panel: aggregation of particles.

In summary, highly crystalline ZIF-8 can be synthesized just by bringing solid ZnCarb and HmeIm in contact in a closed vial. To the best of our knowledge, this is the first example of a MOF synthesized under such undemanding conditions. Moreover, our ZIF-8 demonstrates the highest surface area and pore volume values among self-generated porous materials. This apparently simple reaction does not require additives or mechanical energy input and exhibits a complex sigmoidal kinetics with an initial induction period and an explosive increase in reaction rate. We attribute this behaviour to self-generated H_2_O accumulating in the system and acting as a solvent for HmeIm, thus promoting the reaction. Water added directly to the mixture as well as shaking the reagents increase the reaction rate and minimize the induction period. We believe that this simple, cheap, and environmental-friendly approach can be adapted for the continuous large-scale preparation of material ZIF-8 by extrusion using small amounts of water.

## Author contributions

Investigation, N. G., J. A. V.; SEM/EDX, I. F.; writing and editing, N. G., J. A. V., and F. E.

## Conflicts of interest

There are no conflicts to declare.

## Supplementary Material

RA-012-D2RA00740A-s001
