# Supplementary material for: Mix and wait – a relaxed way for synthesizing ZIF-8
Source: RSC Adv. 2022 Mar 22;12(15):8940–4. doi: 10.1039/d2ra00740a (PMC8985132; doi:10.1039/d2ra00740a)
Supplement: RA-012-D2RA00740A-s001 [file RA-012-D2RA00740A-s001.pdf]

Electronic Supplementary Material (ESI) for RSC Advances.
This journal is © The Royal Society of Chemistry 2022

Supplementary Information

Mix and wait – a relaxed way for synthesizing ZIF-8

Nikita Gugin,[a,b] Jose Antonio Villajos,[a] Ines Feldmann,[a] and Franziska Emmerling*[a,b]

[a] Federal Institute for Materials Research and Testing (BAM), Richard-Willstätter-Str. 11, 12489 Berlin, Germany.

[b] Department of Chemistry, Humboldt Universität zu Berlin, Brook-Taylor-Str., 12489 Berlin, Germany.

1. Materials

The following chemicals were used without further purification: basic zinc carbonate (97 %, Alfa Aesar), zinc acetate dihydrate (>99.5 %, Honeywell Fluka™), zinc nitrate hexahydrate (≥99 %, Roth), zinc hydroxide (98 %, fluorochem), 2-methylimidazole (≥98 %, Roth), Basolite® Z1200 produced by BASF (Aldrich) as reference ZIF-8 material. Water was acquired from a Milli-Q® Direct Water Purification System [18.2 MΩ•cm (25°C)].

2. Synthesis

ZIF-8 was obtained *via* simply mixing powders of 2-methylimidazole (HmeIm) and basic zinc carbonate (ZnCarb) in an HmeIm/Zn molar ratio of 2:1. To increase the area of contact between solid surfaces, the large-crystal HmeIm was ground into a powder. For this, a vertical miller (Pulverisette 23, Fritsch, Germany) with two stainless steel grinding balls (10 mm) were used to yield ca. 2 g of the HmeIm fine powder after 5 min at 50 Hz of milling frequency. The fine powder of ZnCarb was used as purchased. Removal of unreacted HmeIm was performed according to the reported procedure[1] by washing, sonicating (1 min), and centrifugation (10 min) of the as-synthesized powder in 50 mL of ethanol. After three washes, the sample was dried at 60°C overnight.

2.1 Non-shaking experiments

HmeIm (0.36 g, pre-ground) and ZnCarb (0.24 g, as purchased) were placed in a beaker (50 mL) and thoroughly mixed with a spatula for 3 minutes. Several equally prepared mixtures were sealed in 8 mL glass vials and kept undisturbed at ambient conditions for 4, 16, 22, 46 hours, 1 week, and 4 weeks. After the specified elapsed time, the vials were opened and powder X-ray diffraction (PXRD) patterns were quickly recorded. Each experiment was repeated three times to check its reproducibility (Fig. S1). For comparison, a reference mixture was kept in an open vial for one month (Fig. S1).

2.2 Shaking experiments

HmeIm (0.36 g, pre-ground) and ZnCarb (0.24 g, as purchased) were placed in a beaker (50 mL) and thoroughly mixed with a spatula for 3 minutes. Several equally prepared mixtures were sealed in 8 mL glass vials and shook at 800 rpm on the shaker Minishaker MS 2 (IKA, Germany) with circular motion of the agitation table at ambient conditions for 4, 8, 12, 16, 22, and 46 hours. After the specified elapsed time, the vials were opened, and PXRD patterns were quickly recorded. Each experiment was repeated three times to check its reproducibility (Fig. S2).

2.3 Experiment in the dry atmosphere

The reaction in the dry atmosphere was prepared as follows: HmeIm (0.36 g, pre-ground) and ZnCarb (0.24 g, as purchased) were placed in a glove box under dry argon atmosphere. The reagents were thoroughly mixed in a beaker (50 mL) with a spatula for 3 minutes, sealed in 8 mL glass vial, and kept undisturbed for 48 h. After the specified elapsed time, the vial was opened and the PXRD pattern was quickly recorded (Fig. S3).

2.4 Experiments using different precursors

To study the effect of different metal sources, we prepared the following three mixtures: i) HmeIm (0.21 g, pre-ground) and zinc acetate dihydrate (0.29 g, pre-ground), ii) HmeIm (0.18 g, pre-ground) and zinc nitrate hexahydrate (0.32 g, pre-ground), iii) HmeIm (0.31 g, pre-ground) and zinc hydroxide (0.19 g). The first two mixtures react already at mixing, as evidenced by the fluid release. After drying the mixtures the corresponding PXRD patterns were recorded (Fig. S3). The (iii) mixture of HmeIm and zinc hydroxide, in turn, was thoroughly mixed in a beaker (50 mL) with a spatula for 3 minutes, sealed in 8 mL glass vial, and kept undisturbed for 48 h. After the specified elapsed time, the vial was opened and the PXRD pattern was quickly recorded (Fig. S3).

3. Analytical Investigations: Instruments and Methods

3.1 Powder X-ray diffraction (PXRD)

PXRD patterns were collected with Cu Kα radiation (λ = 1.50406 nm) in a Bragg Brentano D8 Advanced diffractometer (Bruker AXS, Germany) equipped with a LYNXEYE XE-T detector. Samples were measured in reflection geometry in a 2ϴ range from 3° to 50° with a step size of 0.02° with spinning setup.

Powder diffraction measurements of the samples prepared under dry argon atmosphere were performed with a D8 Discover diffractometer (Bruker AXS, Karlsruhe, Germany) operated in transmission geometry (Cu-K_{α}1 radiation, λ = 0.154056 nm), equipped with a Lynxeye detector. Samples were prepared in borosilicate glass capillaries (diameter 0.5 mm) and were measured in a 2θ range of 5–50° with a step size of 0.009° and 4 s per step.

3.2 In-situ Raman spectroscopy

Raman measurements were performed on a Raman RXN1™ analyser (Kaiser Optical Systems, France), with a non-contact probe head (working distance of 6 cm, spot size = 1 mm), using an excitation wavelength of l = 785 nm for the collection of Raman spectra every 15 min. For every measurement, five spectra with an acquisition time of 5 s were accumulated. The jar contribution subtraction, baseline correction, and normalization were performed using a script for basic analysis and visualization of in situ Raman monitoring data[2].

Dry ZIF-8 self-formation

To perform the non-shaking synthesis for in-situ Raman monitoring, HmeIm (0.36 g, pre-ground) and ZnCarb (0.24 g, as purchased) were placed in a beaker (50 mL) and thoroughly mixed with a spatula for 3 minutes. The mixture was sealed in the 3.5 mL PMMA jar, which is transparent to Raman laser radiation, and kept undisturbed until the end of the Raman experiment (70 h). To perform the shaking synthesis, the mixture sealed in the 3.5 mL PMMA jar was shaken for 8 h at 50 Hz using Pulverisette 23 vertical miller (Fritsch, Germany).

Water-assisted ZIF-8 self-formation

Two types of water-assisted experiments were performed: In the first kind of experiment, HmeIm (0.36 g, pre-ground) and ZnCarb (0.24 g, as purchased) were placed in a beaker (50 mL) and thoroughly mixed with a spatula for 3 minutes. Then 50 µL of Milli-Q water was added there and mixed 1 min more. After that, the mixture was sealed in the 3.5 mL PMMA jar and kept undisturbed until the end of the Raman experiment (45 h). In the second kind of experiment, HmeIm (0.36 g,

pre-ground) and ZnCarb (0.24 g, as purchased) were placed in a beaker (50 mL) and thoroughly mixed with a spatula for 3 minutes. 50 μL of Milli-Q water was added to the bottom of the 3.5 mL PMMA jar, and the reaction mixture was added to the jar with no further mixing with the water below. The jar was sealed and kept undisturbed until the end of the Raman experiment (48 h).

3.3 Calibration of the Raman signal to calculate the conversion of reagents

To use the Raman spectroscopy for quantitative analysis, a 3-component mixture of solid samples was analysed. Several mixtures of basic zinc carbonate, 2-methylimidazole, and reference ZIF-8 were prepared as follows: HmeIm (1 g, pre-ground) and ZnCarb (0.67 g, as purchased) were placed in a beaker (50 mL) and thoroughly mixed with a spatula for 5 minutes. After that, the material was placed in several glass vials (8 mL) and mixed (3 min, spatula) with the equivalent amount of ZIF-8 to a conversion of 6, 32, 55, 76, and 96% of ZnCarb to ZIF-8, preparing three mixtures for each concentration as standards. Several measurements were performed for each mixture, and the results were processed analogously to those obtained in the in-situ mode using a script for analysis of in situ Raman data[2]. The calibration curve is plotted below, where the conversion of HmeIm was calculated from the ratio between the 1481 cm^{-1} band of HmeIm (I1) and the 1461 cm^{-1} band of ZIF-8 (I2).

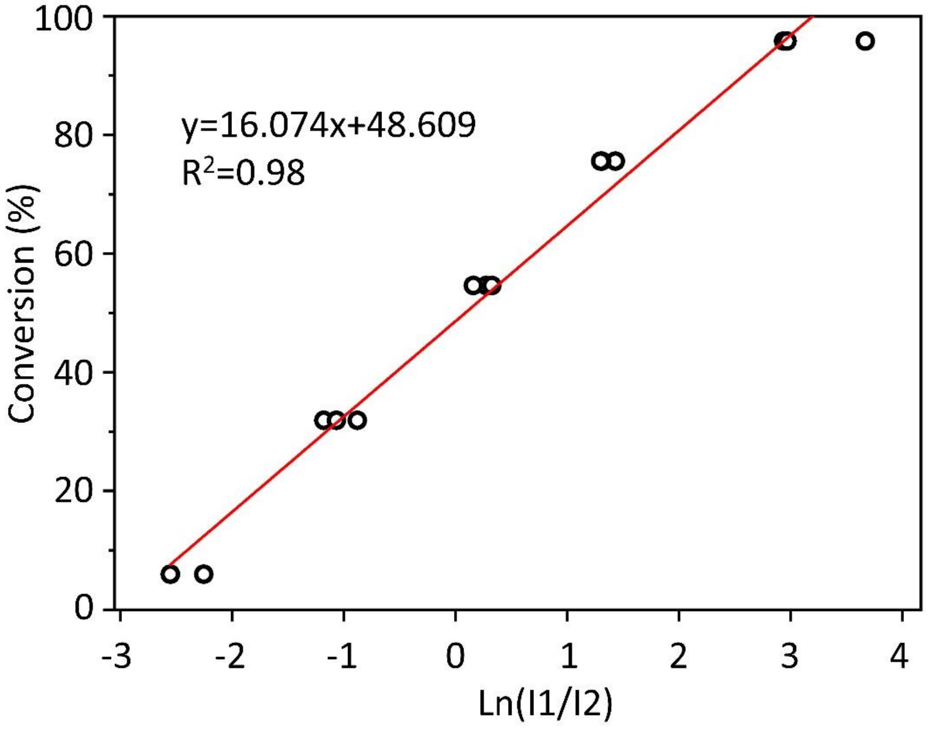

3.4 Thermogravimetry (TGA)

TGA measurements were conducted on a TGA/DSC 3^+ Thermogravimetric Analyzer (Mettler-Toledo, USA) in an atmosphere of air/nitrogen gas mixture (4/5 of air and 1/5 of nitrogen) in the temperature range of 25–600 °C, at a heating rate of 10 °C/min.

3.5 Scanning Electron Microscopy (SEM)

The Scanning Electron Microscopy (SEM) characterization was conducted on an XL 30 ESEM equipped with a tungsten cathode (FEI, Eindhoven, in 2020 electronic upgrade by point electronic GmbH). An Energy-Dispersive X-ray system (EDX, Quantax 200, Bruker Nano GmbH, X Flash 6/60 SDD) was used for the quantitative analyses of the elemental composition. Both, ESEM and EDS investigations, were performed in "high-vacuum mode" of the microscope. The signal was processed with a BackScatter Electron detector (BSE) and a Secondary Electron detector (SE). Prior to the analysis, all samples were coated with carbon. The corresponding EDS spectra were collected at an accelerating voltage of 10 keV and the quantification was performed standardless.

3.6 N_2 adsorption measurements

N_2 adsorption/desorption isotherms were measured at 77 K on a volumetric ASAP 2020 device from Micromeritics in the range of relative pressures p/p_0 from 10^{-4} to 0.996. For analysis, 0.15 g of material were loaded in the analysis probe, followed by thermal activation at 120 °C for 18 hours under a dynamic vacuum to reach a pressure below 10^{-7} mbar. The BET area (A_{BET}) was calculated by following Rouquerol and Llewellyns' rule to select the optimal BET range.[3] The total pore volume (Vp) was calculated from the N_2 uptake at $p/p_0 \approx 0.9$.

4. Results

Fig. **S1** PXRD patterns of the products prepared by keeping the reagent mixtures in open and closed vials at different times with no shaking. The digit after the underscore denotes the number of repetition.

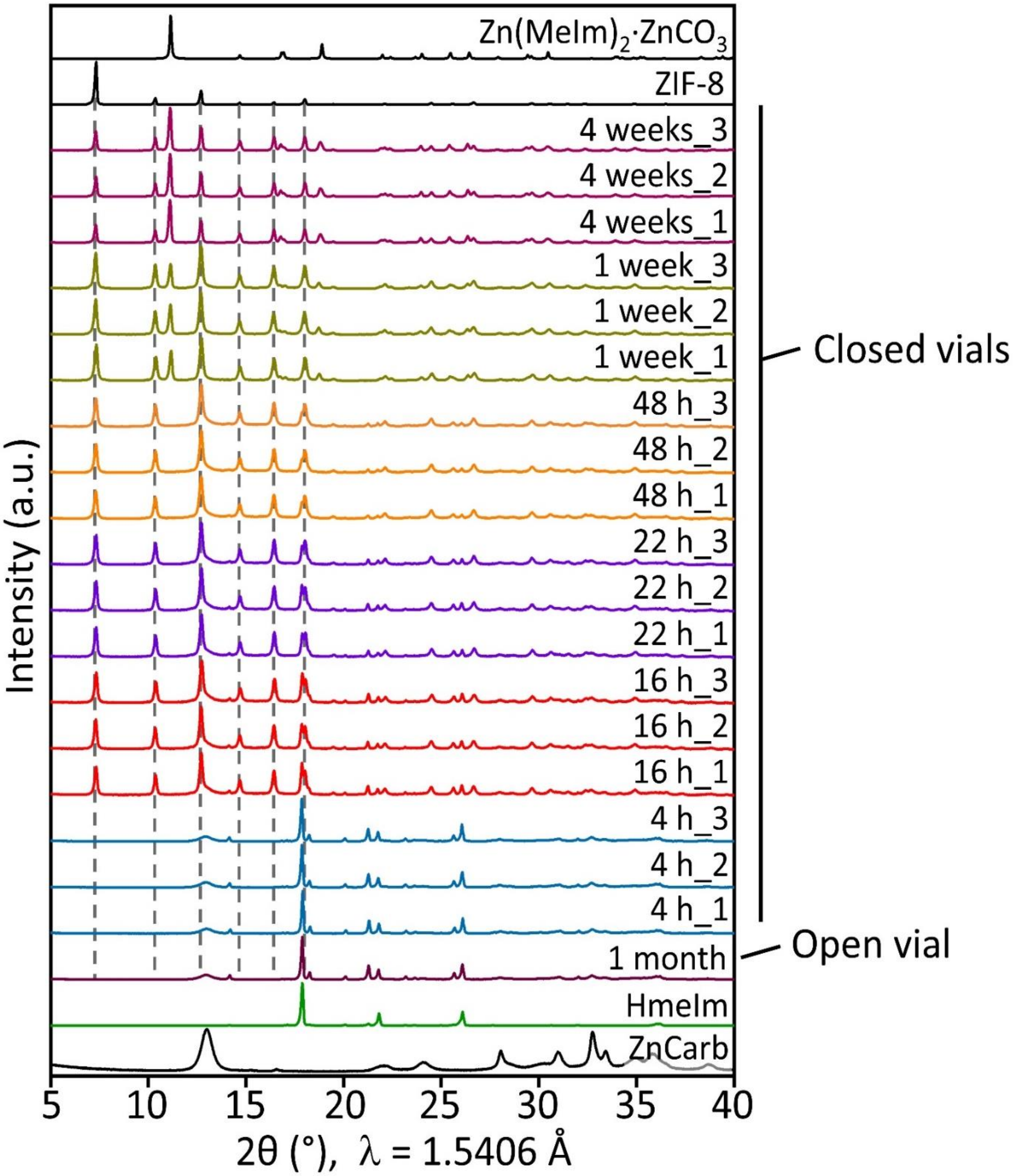

Fig. S2 PXRD patterns of the products prepared by shaking the reagent mixtures at different times in closed vials. The digit after the underscore denotes the number of repetition.

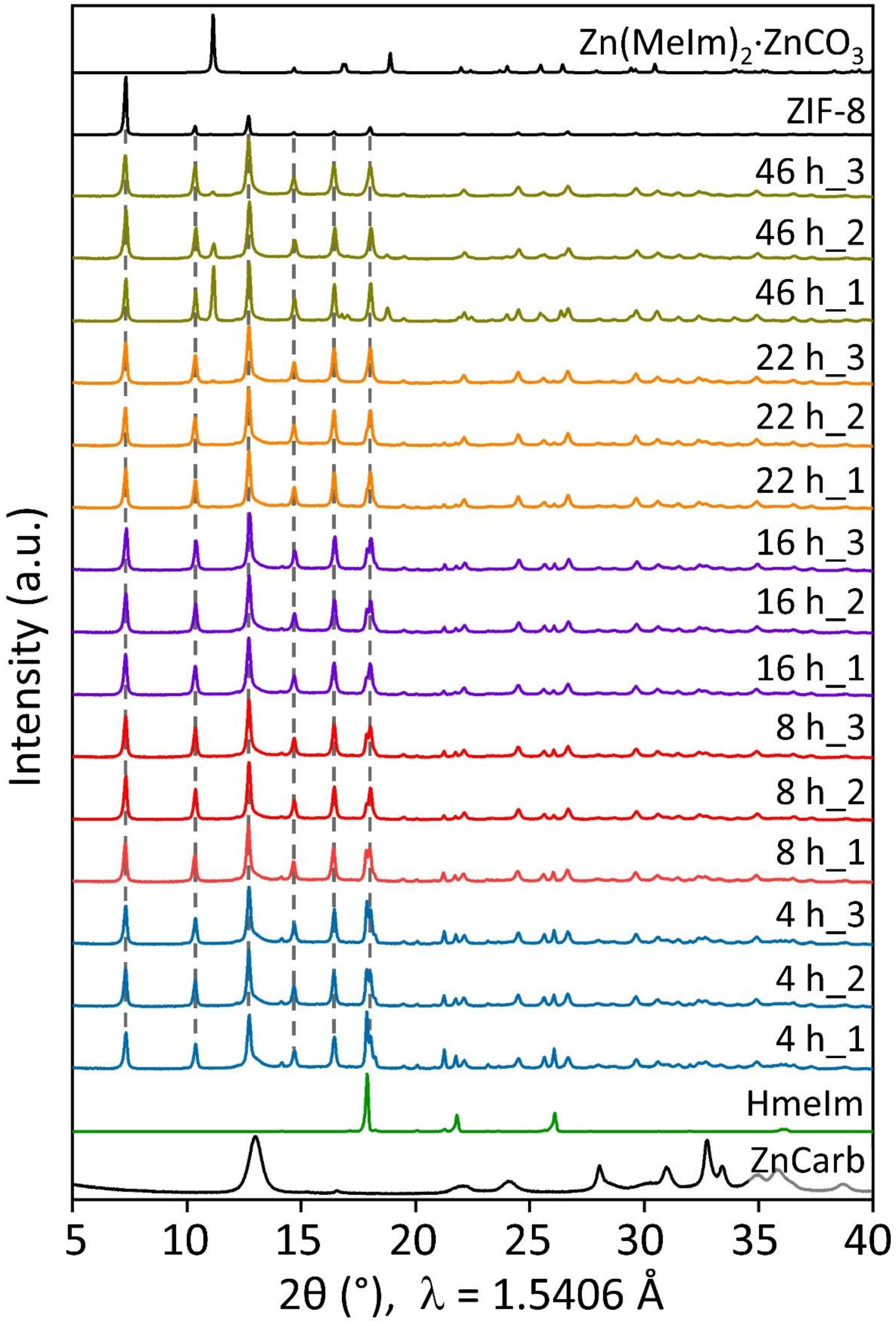

Fig. S3 PXRD patterns of the products prepared from different zinc sources: zinc hydroxide (purple), zinc nitrate hexahydrate (blue), zinc acetate dihydrate (brown). Green pattern corresponds to the product obtained from ZnCarb and HmeIm mixture kept under dry argon atmosphere for 48 h.

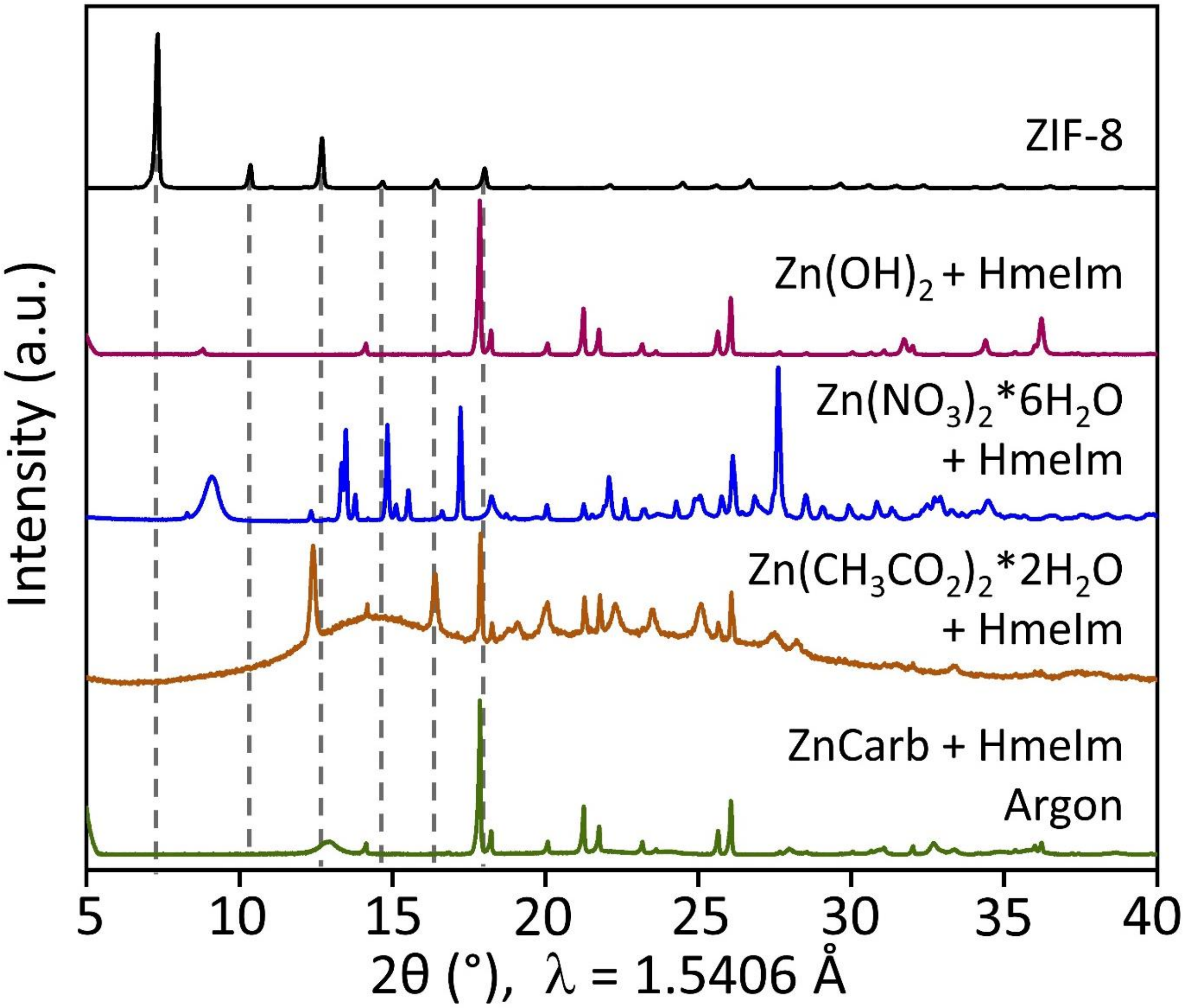

Fig. **S4** Thermogravimetric curves of as-made and ethanol-washed products.

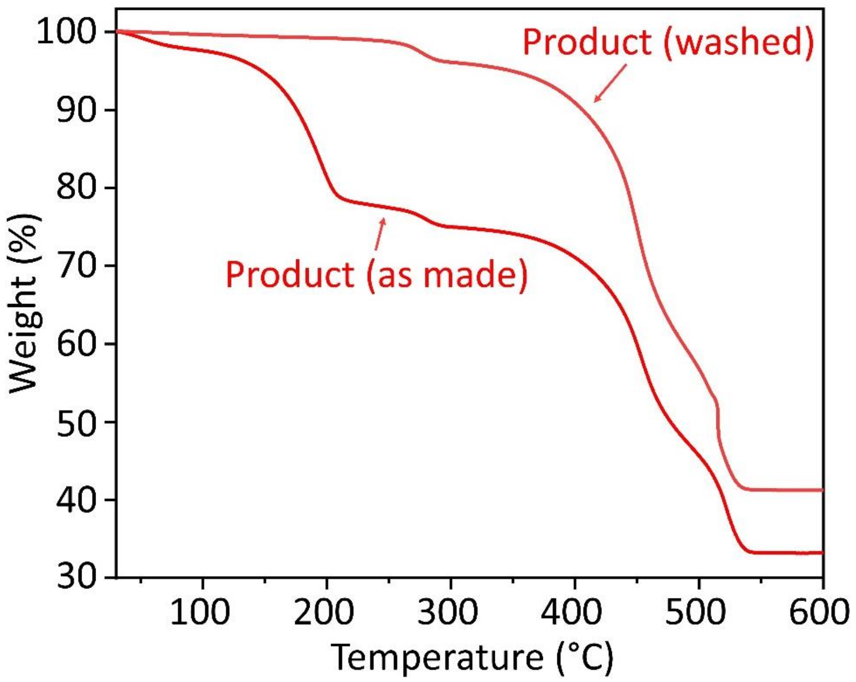

Fig. **S5** SEM pictures of the as-synthesized product using BSE (left) and SE (right) detectors. Red stars indicate the spots where EDX spectra were recorded

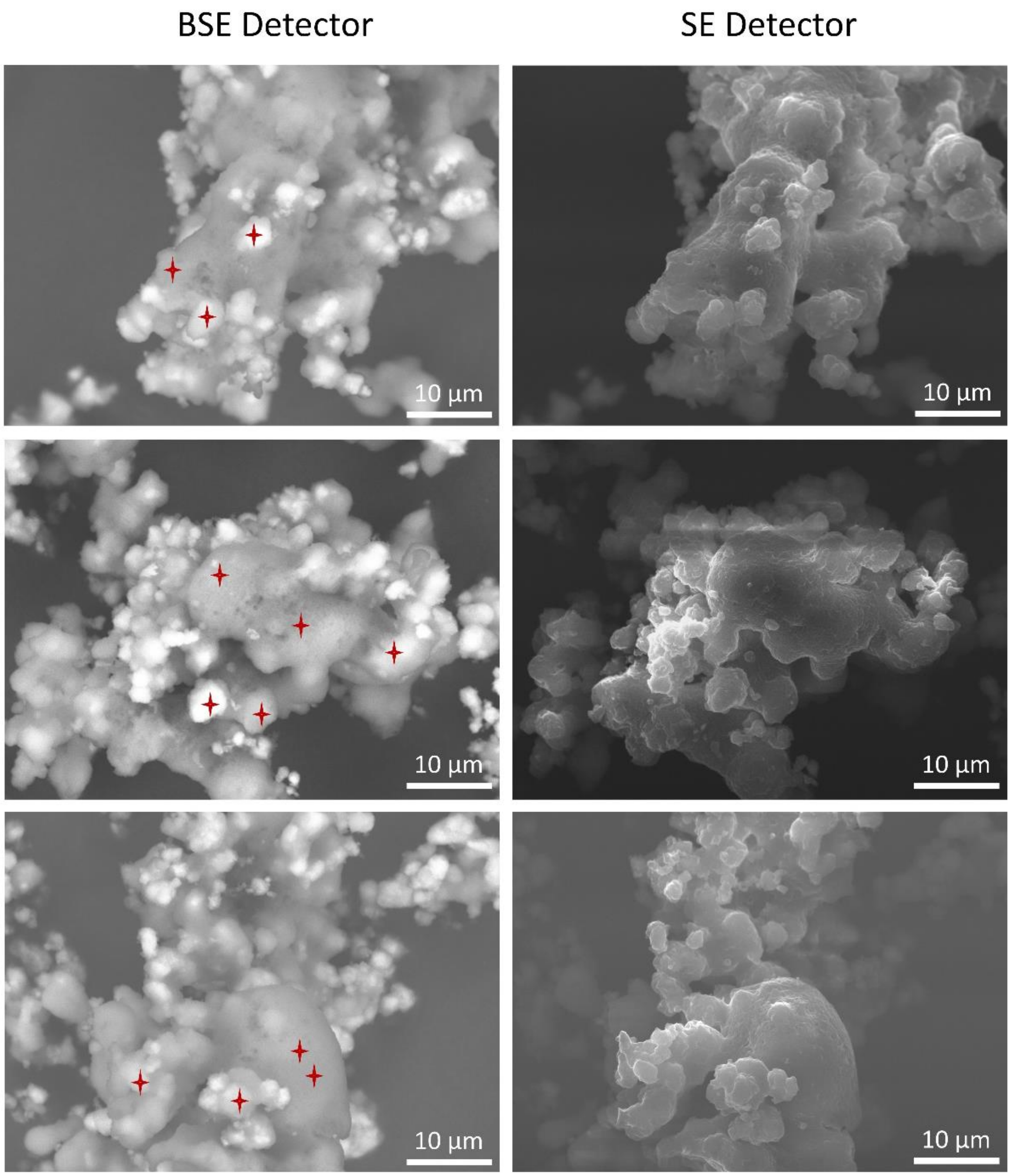

Fig. **S6** SEM pictures of the as-synthesized products after shaking the mixtures for 4, 16 and 46 h (taken with BSE detector).

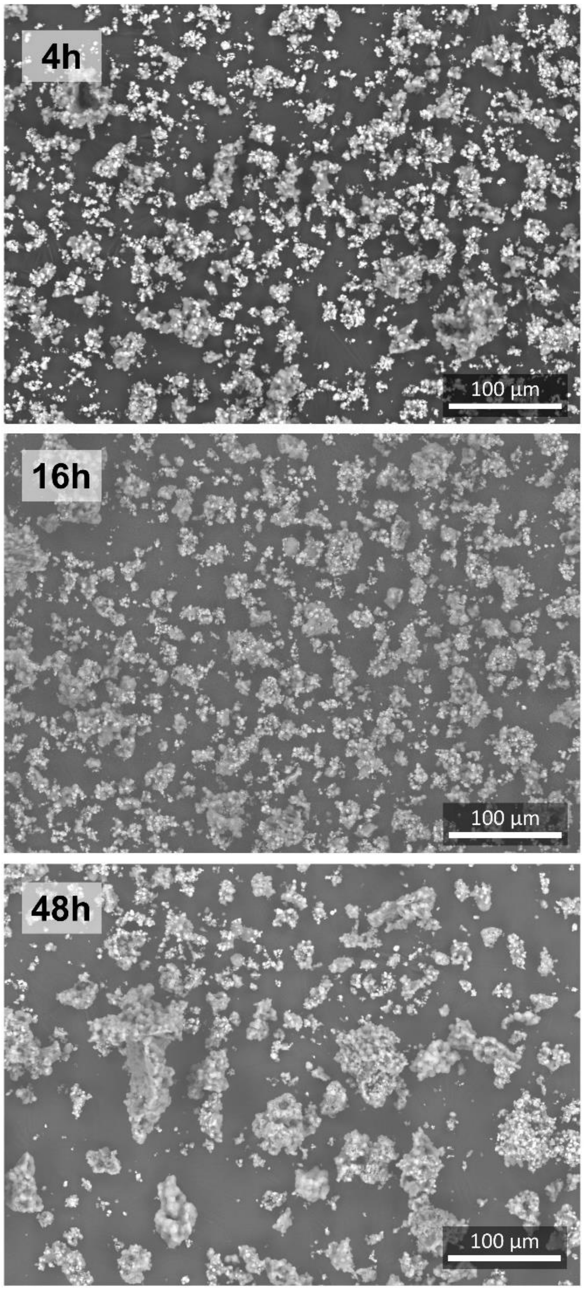

Table S1 The EDX evaluation of the as-synthesized ZIF-8 in the dark and bright regions compared with the theoretical values for pure ZIF-8 and ZnCarb.

Ratio	Dark spots	Bright spots	ZIF-8	$[ZnCO_3]_2 \cdot [Zn(OH)_2]_3$
Zn/N	0.2	0.5	0.25	-
Zn/O	2.0	0.5	-	0.4
N/O	11.5	1.7	-	-

References

1. Taheri, M.; Di Bernardo, I.; Lowe, A.; Nisbet, D. R.; Tsuzuki, T., Green Full Conversion of ZnO Nanopowders to Well-Dispersed Zeolitic Imidazolate Framework-8 (ZIF-8) Nanopowders via a Stoichiometric Mechanochemical Reaction for Fast Dye Adsorption. *Crystal Growth & Design* **2020,** *20* (4), 2761-2773.
2. Lukin, S.; Uzarevic, K.; Halasz, I., Raman spectroscopy for real-time and in situ monitoring of mechanochemical milling reactions. *Nat Protoc* **2021,** *16* (7), 3492-3521.
3. Thommes, M.; Kaneko, K.; Neimark, A. V.; Olivier, J. P.; Rodriguez-Reinoso, F.; Rouquerol, J.; Sing, K. S. W., Physisorption of gases, with special reference to the evaluation of surface area and pore size distribution (IUPAC Technical Report). *Pure and Applied Chemistry* **2015,** *87* (9-10), 1051-1069.
